# Autoimmune Hemolytic Anemia After Cyanocobalamin Replacement in a Patient With a Previous Diagnosis of Pernicious Anemia: A Case Report

**DOI:** 10.7759/cureus.10797

**Published:** 2020-10-05

**Authors:** Leonardo Mejia Buritica, Jesus Zapata Alvarez, Lissette Vergara Quintero, Juan Pablo Villegas Molina, José Domingo Torres Hernandez

**Affiliations:** 1 Department of Hematology, Universidad de Antioquia, Medellin, COL; 2 Department of Internal Medicine, Universidad de Antioquia, Medellin, COL

**Keywords:** autoimmune hemolytic anemia (aiha), pernicious anemia, direct antiglobulin test, coombs test

## Abstract

Pernicious anemia (PA) is associated with other autoimmune diseases, such as hypothyroidism, type 1 diabetes mellitus (DM1), Addison's disease, and vitiligo. The association between PA and autoimmune hemolytic anemia (AIHA) is rare, with less than 30 cases reported in the literature. In this paper, we report a case of a patient with a confirmed diagnosis of PA, who, six months after starting treatment with cyanocobalamin, presented with severe hemolysis with a positive direct antiglobulin test (DAT) for warm antibodies; the patient responded well to glucocorticoid treatment. AIHA in PA patients can be triggered by cyanocobalamin replacement due to the expression of membrane antigens by mature red blood cells entering into the peripheral circulation. This association should be considered because these patients, in addition to cyanocobalamin replacement, will require immunosuppressive treatment, usually with glucocorticoids.

## Introduction

Vitamin B12 deficiency is a common cause of megaloblastic anemia, and it occurs in more than 16% of patients with macrocytosis [1-2]. The prevalence increases with age, being more frequent in patients older than 60 years, but it can affect the younger population as well [3]. There are multiple causes of vitamin B12 deficiency, with pernicious anemia (PA) being one of the most prevalent [4]. PA is a disease of autoimmune origin in which antibodies attack the parietal cells of the stomach or intrinsic factor, and presents as atrophic gastritis, anemia, and neurological manifestations [5]. PA is associated with other autoimmune diseases, such as hypothyroidism, type 1 diabetes mellitus (DM1), Addison's disease, and vitiligo [6].

The association between PA and autoimmune hemolytic anemia (AIHA) is rare, with only a few cases described in the literature [2]. In this paper, we present the case of a patient with a confirmed diagnosis of PA, who developed severe AIHA with a positive direct antiglobulin test (DAT) for warm antibodies after the initiation of cyanocobalamin replacement.

## Case presentation

A 56-year-old man, with no significant medical history, presented to the emergency department with a two-month history of asthenia, adynamia, and weight loss. Two hours prior to the admission, he had felt abdominal pain in the right upper quadrant. The physical examination was remarkable for tenderness in the right upper quadrant and generalized mucocutaneous pallor. Acute cholecystitis was diagnosed, and laparoscopic cholecystectomy was performed without complications.

Laboratory tests on admission showed macrocytic anemia, vitamin B12 deficiency, and low reticulocytes (Table 1). Intrinsic factor antibodies were positive. A digestive endoscopy was performed, which revealed macroscopic atrophic gastritis. The gastric biopsy showed chronic atrophic gastritis, decreased parietal cells, and reduction of fundic glands. The diagnosis of PA was confirmed, and treatment was started with cyanocobalamin 1 milligram intramuscular (IM) daily for two weeks and then 1 milligram IM monthly, which led to the resolution of anemia and improvement of symptoms.

**Table 1:** Laboratory tests DAT: direct antiglobulin test; HIV: human immunodeficiency virus; HBsAg: hepatitis B surface antigen

Test	Reference range	Initial value	Six months later	Two weeks after glucocorticoid treatment
Hemoglobin	14–18 g/dL	9.7 g/dL	5.4 g/dL	8.4 g/dl
Hematocrit	42–50%	26.4%	16.7%	23.8%
Mean corpuscular volume	80–98 fL	115.3 fL	127.5 fL	110 fL
Platelet count	150,000–450,000/microL	313,000/microL	387,000/microL	430,000/microL
Leukocyte count	4,000–11,000/microL	9,850/microL	11,150/microL	9,000/microL
Reticulocyte count	0.5–1.5%	1.32%	22.8%	
Total bilirubin	0.3–1 mg/dL	2.59 mg/dl	2.48 mg/dl	1.32 mg/dl
Direct bilirubin	0.1–0.3 mg/dL	1.22 mg/dl	0.96 mg/dl	0.59 mg/dl
Vitamin B12	200–800 pg/mL	93.59 pg/mL	412 pg/mL	
Lactate dehydrogenase	80–225 units/L	526 units/L	860 units/L	424 units/L
Ferritin	24–336 ng/mL		425 ng/L	
DAT	Negative	Negative	Positive 4+	
Intrinsic factor antibodies	≤10.0 units/mL	47.6 units/mL		
HIV test	Negative		Negative	
Non-treponemal test	Negative		Negative	
Hepatitis C antibodies	Negative		Negative	
HBsAg	Negative		Negative	

Six months later, the patient presented again to the emergency department with a two-week history of asthenia, adynamia, dizziness, exertional dyspnea, and headache. The physical examination was remarkable for a temperature of 38 degrees Celsius, generalized mucocutaneous paleness, and jaundice. The laboratory test revealed severe anemia (Table 1), and red blood cell transfusion was ordered. Nevertheless, the blood transfusion could not be performed due to positive cross matches with all the units available in the blood bank.

**Figure 1:**
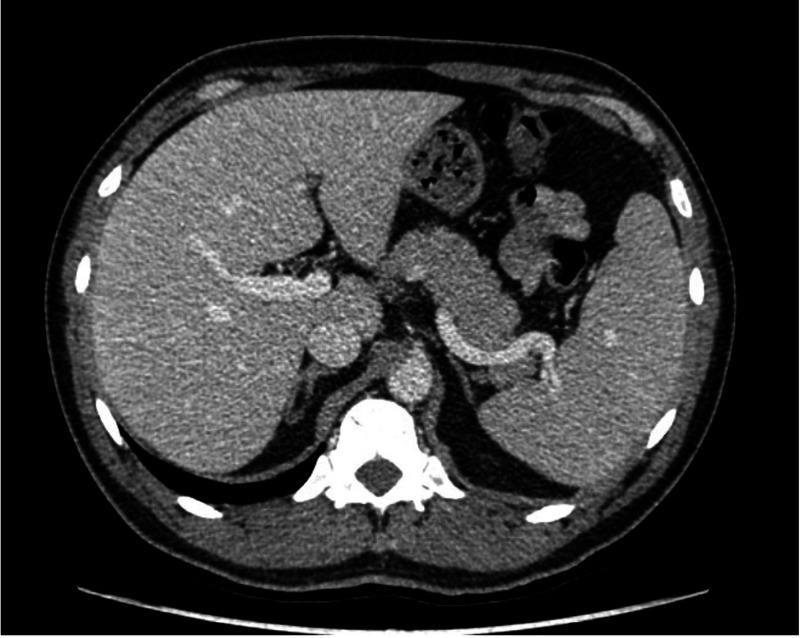
Computed tomography scan showing an enlarged liver and spleen

A contrasted CT of the chest and abdomen was performed, which showed hepatomegaly and mild splenomegaly (Figure 1) without other significant findings; and a bone marrow aspiration and biopsy were performed, which reported erythroid hyperplasia without other alterations. Also, there was no evidence of lymphoid neoplasia on flow cytometry.

**Figure 2:**
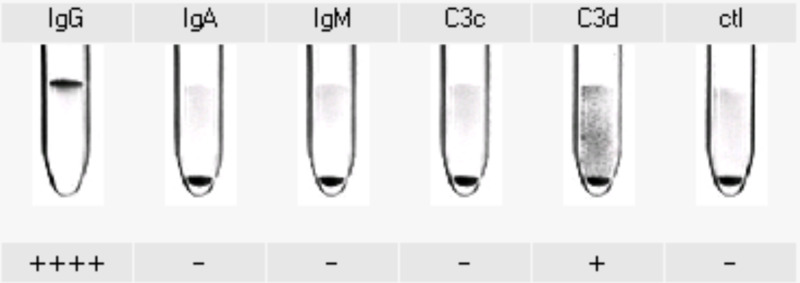
Monospecific direct antiglobulin test positive for IgG and C3d IgG: immunoglobulin G; IgA: immunoglobulin A; IgM: immunoglobulin M; C3 complement fraction 3; ctl: control

Vitamin B12 levels were normal, and DAT was strongly positive for warm antibodies (Figure 2). A diagnosis of warm-AIHA was established and treatment with prednisolone at 1 mg/kg/day was started.

Two weeks after the start of treatment with glucocorticoids, lactate dehydrogenase (LDH) levels decreased, hemoglobin improved, and the patient was discharged with oral prednisolone therapy, with an outpatient reduction dose plan. Cyanocobalamin replacement was continued.

## Discussion

Anemia is a frequent clinical finding in daily medical practice and can be caused by multiple etiologies [7]. Vitamin B12 deficiency is a frequent cause of megaloblastic anemia [1], and PA is one of the most important causes of this condition. The association between PA and other autoimmune diseases is well recognized [6]; however, very few cases of coexistence between PA and AIHA have been reported in the literature [2]. To our knowledge, less than 30 cases have been reported in the literature of patients with a diagnosis of both diseases, and it is more frequent in middle-aged women with other autoimmune diseases [2].

AIHA is a rare entity, with an approximate incidence of one to three cases per 100,000 people/year [8]; it is characterized by the destruction of red blood cells mediated by autoantibodies directed against antigens present in their membrane [9]. This disease may appear in isolation, also called primary AIHA, or it can be associated with other autoimmune diseases. AIHA could also be associated with primary immunodeficiencies, infectious diseases, and neoplastic disorders [10]. In our case, no other autoimmune or neoplastic diseases were identified. However, in other cases described in the literature, coexistence with other autoimmune diseases has been found, such as hypothyroidism, systemic lupus erythematosus, alopecia areata, vitiligo, and rheumatoid arthritis [2].

From a serological point of view, various forms of AIHA have been described. The most common form is produced by immunoglobulin G (IgG) type antibodies that bind to red blood cells at 37 degrees Celsius, and mainly cause extravascular hemolysis [9]. This form is called warm-AIHA and accounts for 65-70% of AIHA cases [10].

The second most common form is called cold AIHA and accounts for 20-25% of cases [10]. This type of hemolytic anemia is mediated by immunoglobulin M (IgM) type antibodies that bind to membrane antigens at temperatures lower than body temperature, optimally 4 degrees Celsius in vitro [8]. The responsible immunoglobulin can be polyclonal or monoclonal, the latter present in lymphoproliferative neoplasms such as chronic lymphocytic leukemia, Waldenstrom macroglobulinemia, and other non-Hodgkin lymphomas [11].

For the diagnosis of AIHA, the DAT test (also known as the Coombs test) is required, which is positive in most cases (only 5% of patients with AIHA are DAT-negative) [12]. When DAT is positive in a patient with overt hemolytic anemia with increased reticulocytes, indirect hyperbilirubinemia, increased LDH, and low haptoglobin, the diagnosis of AIHA is highly likely [13]. However, DAT can also be positive in individuals without hemolysis, being detected in 2% of the normal population [14].

Two types of DAT are available, polyspecific and monospecific. Polyspecific DAT simultaneously detects IgG and complement fraction 3 (C3), and it is the screening test for suspected AIHA [15]. If polyspecific DAT is positive, then monospecific DAT should be performed, which contains specific antibodies that bind to IgG, immunoglobulin A (IgA), IgM, and complement fractions C3c and C3d. This test classifies AIHA as warm antibodies (IgG +/- C3) or cold antibodies (IgM or C3) [16]. Mixed AIHA has also been described in cases expressing both warm and cold antibodies [17].

In patients with PA, DAT may be transiently positive when there is no adequate replacement of cyanocobalamin. These cases can be easily differentiated from AIHA since they present with low reticulocytes, and anemia improves rapidly after the initiation of cyanocobalamin replacement, and DAT becomes negative with treatment [18-19]. On the other hand, patients with AIHA usually present with increased reticulocytes and anemia does not improve until the start of immunosuppressive treatment, usually with glucocorticoids. Prophylactic folic acid is also indicated because active hemolysis can consume folate and cause megaloblastosis [2]. In our case, the initial DAT was negative, and the reticulocytes increased after cyanocobalamin replacement. Furthermore, the hemolysis improved with glucocorticoids, which clearly demonstrates the autoimmune origin of red blood cell destruction.

Some cases in which AIHA starts after cyanocobalamin replacement have also been reported. Vucelić et al. reported a patient with vitamin B12 deficiency, who, eight days after cyanocobalamin replacement, presented with worsening of hemolysis and the DAT became positive; treatment with glucocorticoids was started with improvement of hemolysis [20]. Our case is remarkable because the clinical manifestations of hemolysis began six months after cyanocobalamin replacement.

The mechanisms that explain autoimmune hemolysis in patients with PA are not clear. It is proposed that the treatment of PA causes the mature red blood cells to express membrane antigens and enter into peripheral circulation where they are attacked by self-reactive antibodies, triggering autoimmune hemolysis [20]. However, this will only occur in individuals with a previously established background of autoimmunity.

## Conclusions

PA is an autoimmune disease clearly associated with other antibody-mediated diseases. The association between PA and AIHA is rare, with only a few cases reported in the literature. Our case was remarkable because of the manifestation of AIHA six months after the treatment with cyanocobalamin in a patient with PA. We hope this paper will prompt clinicians to consider the diagnosis of AIHA in any patient with PA who presents with worsening anemia, high reticulocytes, and positive DAT after starting treatment with cyanocobalamin. In these cases, immunosuppressive treatment, mainly with glucocorticoids, must be started quickly.
